# Acute kidney injury in critically ill newborns: What do we know? What do we need to learn?

**DOI:** 10.1007/s00467-008-1060-2

**Published:** 2009-02-01

**Authors:** David J. Askenazi, Namasivayam Ambalavanan, Stuart L. Goldstein

**Affiliations:** 1 Division of Pediatric Nephrology, University of Alabama at Birmingham, 1600 7th Ave So., ACC 516, Birmingham, AL 35233 USA; 2 Division of Perinatal Medicine, University of Alabama at Birmingham, Birmingham, AL USA; 3 Division of Pediatric Nephrology, Baylor College of Medicine, Houston, TX USA

**Keywords:** Renal failure, Outcomes, Biomarker, Neonate, Premature, Chronic

## Abstract

Outcomes in critically ill neonates have improved over the past three decades, yet high residual mortality and morbidity rates exist. Acute kidney injury (AKI) is not just an innocent by-stander in the critically ill patient. Research on incidence and outcomes of AKI in the critically ill neonatal population is scarce. The objective of this publication is to (a) review original articles on the short- and long-term outcomes after neonatal AKI, (b) highlight key articles on adults and children with AKI in order to demonstrate how such insights might be applied to neonates, and (c) suggest clinical research studies to fill the gaps in our understanding of neonatal AKI. To date, observational studies suggest high rates of AKI and poor outcomes in critically ill neonates. Neonates with AKI are at risk of developing chronic kidney disease and hypertension. Large prospective studies are needed to test definitions and to better understand risk factors, incidence, independent outcomes, and mechanisms that lead to poor short- and long-term outcomes. Early biomarkers of AKI need to be explored in critically ill neonates. Infants with AKI need to be followed for sequelae after AKI.

## Introduction

Advancements in neonatal–perinatal medicine have improved the survival rates of critically ill neonates, yet residual mortality and morbidity rates are significant [1]. Acute kidney injury (AKI) is a complex disorder with clinical manifestations ranging from mild dysfunction to complete anuric kidney failure. Our understanding of the epidemiology of neonatal AKI is based on small single-center studies that usually focus on a subset of the neonatal population. Nonetheless, the available data suggest that the incidence of AKI in asphyxiated neonates is high, that non-oliguric AKI is common, and that AKI portends poor outcomes [2–6]. Few articles on other critically ill infants are limited to retrospective cross-sectional studies or prospective studies with poor definitions of AKI [5, 7, 8] (reviewed below). The independent impact of AKI on outcomes has not been explored, probably due to limitations of sample size and lack of a consistent definition.

Novel urinary biomarkers can diagnose AKI within hours of an insult have been discovered. The original experience with several of these biomarkers occurred in neonates who required cardiopulmonary bypass surgery. Studies to detect AKI in non-cardiac critically ill neonates have not been performed. These biomarkers will change our approach to the diagnosis AKI and, hopefully, lead to better preventative and therapeutic interventions which will improve outcomes of critically ill neonates.

Rodriguez et al. [9] showed that the extra-uterine environment is not optimal for glomerular development, an effect which could be exaggerated in those with AKI. Mounting evidence shows that premature infants are at risk of hypertension, chronic kidney disease and metabolic syndrome. In addition, after hospitalization for AKI, adults and children are at risk of death or chronic kidney disease (CKD). Thus, premature infants with AKI are at risk for CKD [4, 10].

Publications on neonatal AKI and key pediatric and adult AKI will be used to explain our current understanding of the diagnosis, incidence, outcomes, and potential role of biomarkers in early detection of neonatal AKI. Research priorities to fill the gaps in our current understanding will be suggested.

## Definition of acute kidney injury

Acute kidney injury is a complex disorder that occurs with variable severity and in many clinical scenarios. Until recently, lack of universally recognized definition of AKI limited our ability to compare studies, predict clinical courses, and improve outcomes. In 2004, nephrology and critical care groups recognized these limitations and proposed an empiric working AKI definition [11] (Table 1): The RIFLE (risk, injury, failure, loss, and end-stage renal disease) classification has been studied extensively in several adult populations whereby a change in renal function and/or the presence of oliguria fulfills criteria [12, 13]. Using these AKI classification definitions, studies of critically ill pediatric and adult patients consistently show that AKI has an independent impact upon survival after correction for co-morbidities, complications and severity of illness [14, 15]. These data suggest that patients may not only die with kidney failure but that this dysfunction causes functional and transcriptional changes in the lung and other organs that ultimately lead to poor outcomes [13]. These studies have not been reproduced in critically ill neonatal populations.

**Table 1:** Proposed working definitions for the classification of AKI in adults and children. In all three classifications either creatinine or urine output criteria suffice in staging (*AKIN* Acute Kidney Injury Network, *Cr* creatinine, *GFR* glomerular filtration rate, *eCCl* estimated creatinine clearance, *SCr* serum creatinine, *ESRD* end-stage renal disease)

Adult					Pediatric		
AKIN		AKIN/RIFLE	RIFLE		pRIFLE		
Stage	Serum Cr	Urine output	Class	Serum Cr or GFR	Class	eCCl by Schwartz formula	Urine output
I	↑ SCr >0.3 mg/dl or ↑ SCr >150–200% from baseline	<0.5 ml/kg per hour × 6 h	Risk	↑SCr by 150% or GFR decrease by 25%	Risk	eCCl decrease by 25%	<0.5 ml/kg per hour × 8 h
II	↑ SCr to >200-300% from baseline	<0.5 ml/kg per hour > 12 h	Injury	↑ SCr by 200% or GFR decrease by 50%	Injury	eCCl decrease by 50%	<0.5 ml/kg per hour × 16 h
III	↑ SCr of >300% from baseline or SCr > 4.0 mg/dl with an acute rise of at least 0.5 mg/dl	<0.3 ml/kg per hour >24 h or anuria for >12 h	Fail	↑ SCr by 300% or SCr >4.0 mg/dl with acute rise of 0.5 mg/dl or GFR decrease by >75%	Fail	eCCl decrease by 75% or <35 ml/min per 1.73 m^2^ body surface area	<0.3 ml/kg per hour for 24 h or anuric for 12 h
			Loss	Failure > 4 weeks	Loss	Failure > 4 weeks	
			ESRD	Failure >3 months	ESRD	Failure >3 months	

AKIN classification: an abrupt (within 48 h) reduction in kidney function required (adapted with permission [16])

RIFLE staging: R, I and F represent increasing stages of AKI (reproduced with permission [11])

pRIFLE eCCl based on Schwartz formula (reproduced with permission [17])

In March 2007 the Acute Kidney Injury Network (AKIN), a collaborative group of investigators from all major critical care and nephrology societies, proposed a staging system which use mild AKI (stage 1), moderate AKI (stage 2) and severe AKI (stage 3) in a way similar to the risk, injury and failure categories in RIFLE [16]. The major changes from RIFLE include decreasing the change in serum creatinine (SCr) threshold (0.3 mg/dl) to be classified as mild AKI, and classifying anyone on dialysis as having class 3 AKI (Table 1). In addition, the AKIN criteria include a time requirement to denote the acute changes in kidney function, where changes need to be abrupt: within 48 h (Table 1).

In children, Akcan-Arikan et al. [17] proposed a modified pediatric RIFLE (pRIFLE) classification in which similar criteria were used for pediatrics. The main difference is a lower cutoff SCr to achieve the F category to reflect that, because children have a lower baseline SCr, a SCr of 4.0 mg/dl is not needed to cause severe dysfunction (Table 1).

These classification systems to define AKI in neonatal populations have not been performed. Large prospective studies to test these classification models in neonatal AKI are greatly needed to better understand the impact of AKI on outcomes.

## Diagnosis of acute kidney injury

Despite these working classification systems, the diagnosis of AKI is problematic, as current diagnoses rely on two functional abnormalities: functional changes in serum creatinine [marker of glomerular filtration rate (GFR)] and oliguria. Both these are late consequences of injury and not markers of the injury itself. The ideal marker to detect AKI should be up-regulated shortly after an injury and be independent of GFR level. SCr is the most common method to monitor renal function and to diagnose AKI, but it has significant shortcomings including:

- SCr concentrations may not change until 25–50% of the kidney function has already been lost, and, thus, it may take days before a significant rise in SCr is seen.
- At lower GFR, SCr will overestimate renal function due to tubular secretion of creatinine.
- SCr varies by muscle mass, hydration status, age and gender.
- Different methods (Jaffe reaction vs enzymatic) produce different values, and medications and bilirubin can affect SCr measurements by the Jaffe method [18, 19].
- Once a patient receives dialysis, SCr can no longer be used to assess kidney function, since SCr is easily dialyzed.

**Fig. 1:**
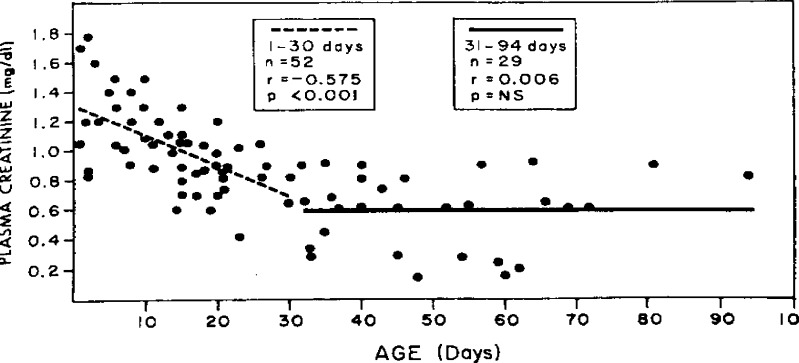
Plasma creatinine in neonates (reproduced with permission from [23])

Additional problems with using SCr as a measure of AKI specific to neonates include:

- SCr in the first few days of the neonate’s life reflects the mother’s and not the infant’s renal function.
- Normal nephronogenesis in healthy term infants begins at 8 weeks of gestation and continues until 34 weeks of gestation, at which time the number of nephrons, 1.6–2.4 million, approximates that of an adult [20]. Dependent on the degree of the neonate’s prematurity, GFR steadily improves from 10–20 ml/min per 1.73 m^2^ body surface area during the first week of life to 30–40 ml/min per 1.73 m^2^ by 2 weeks after birth, concomitant with alterations in renal blood flow. Thereafter, GFR improves steadily over the first few months of life [21] (summarized by Schwartz et al. [22]; Table 2).
- Overall GFR in term and preterm infants is very low, and there is a very wide distribution of normal serum creatinine values, which vary greatly, dependent on level of prematurity and age [23] (Fig. 1).
- Bilirubin levels in premature infants are normal at birth, rise in the first several days and return to normal after a few weeks. If the Jaffe method of SCr is used, this may have tremendous impact on the interpretation of SCr [18].

**Table 2:** Inulin clearance GFR in healthy premature infants (adapted with permission from [22])

Age	Clearance (ml/min per 1.73 m^2^)
Pre-term infants	
1–3 days	14.0 ± 5
1–7 days	18.7 ± 5.5
4–8 days	44.3 ± 9.3
3–13 days	47.8 ± 10.7
1.5–4 months	67.4 ± 16.6
Term infants	
1–3 days	20.8 ± 5.0
4–14 days	36.8 ± 7.2
1–3 months	85.3 + 35.1
4–6 months	87.4 ± 22.3
7–12 months	96.2 + 12.2
1–2 years	105.2 ± 17.3

Owing to the challenges outlined above, our ability to define AKI reliably is very difficult in the neonatal population. The creation of AKI definitions, using early injury biomarkers (in addition to functional markers such as GFR and urine output) which can ultimately predict morbidity and mortality rates is of paramount importance. Until well-designed clinical research studies on AKI definitions have been completed, our ability to recognize and design studies on prevention and treatment in neonates with AKI will be very difficult.

## Epidemiology of neonates with acute kidney injury

**Fig. 2:**
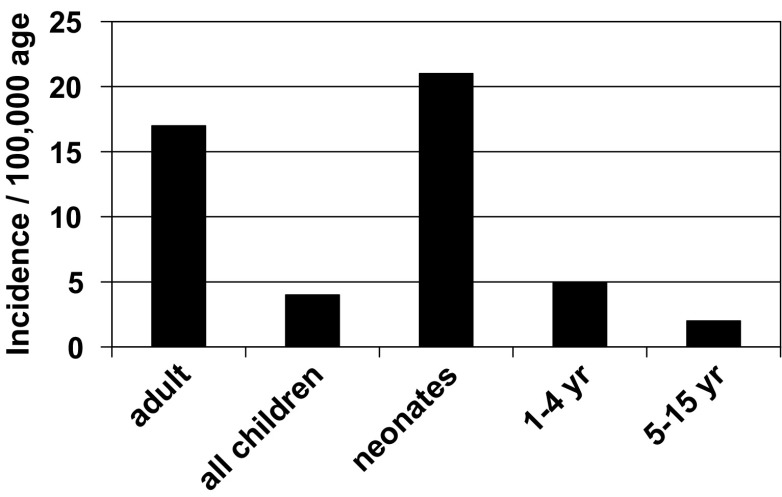
Age-related comparative yearly incidence of AKI (adapted with permission from [25])

Critically ill neonates are at risk of having AKI, as they are commonly exposed to nephrotoxic medications and have frequent infections that lead to multi-organ failure [4]. The exact incidence of neonatal AKI is unknown, but it is likely higher than is reported, as infants very commonly have non-oliguric renal failure. In addition, most studies that describe neonatal AKI use high levels of serum creatinine or need for dialysis to define AKI, which may miss a significant number of infants according to the currently proposed definitions in adults and pediatric populations (Table 1) [5, 24]. Moghal et al. [25] suggested that, relative to other critically ill populations, neonatal AKI has the highest incidence (Fig. 2). Published studies estimate that the incidence of AKI in critically ill neonates is between 8% and 24% and that mortality rates are between 10% and 61% [4].

Most studies on neonates with AKI describe term infants with asphyxia at the time of birth (Table 3). Hypoxic-ischemic encephalopathy (HIE) accounts for 23% of the 4 million neonatal deaths globally and high rates of disability [27]. Three different observational studies describe the incidence of AKI in this subset of critically ill newborns. Karlowicz and Adelman [6] compared term infants with severe asphyxia (according to the Portman asphyxia morbidity scoring system [28]) with similar infants with moderate asphyxia scores. They found that AKI (SCr >1.5 mg/dl) occurred in 20/33 (66%) of infants with severe asphyxia compared with 0/33 (0%) in those with moderate asphyxia. In a case–control analysis (matching for gestational age and birth weight in otherwise healthy newborns) Aggarwal et al. [3] showed that the incidence of AKI in infants with 5 min Apgar scores ≤6 was 56% vs 4% in controls. They noted that clinical markers of asphyxia were better predictors of adverse outcomes than were renal function tests. Similarly, Gupta et al. [2] found a 47% incidence of AKI and 14.1% mortality in infants with Apgar scores ≤6. All these studies report over 50% of AKI cases to be non-oliguric, which highlights the insensitivity of oliguria to predict AKI in neonates.

**Table 3:** Original articles on AKI in neonates (*GA* gestational age, *BUN* blood urea nitrogen, *NICU* neonatal intensive care unit, *UOP* urine output, *DIC* disseminated intravascular coagulation)

Author	Type of study	Cases	Controls	Definition of AKI	Findings	Comments
Karlowicz and Adelman (1995)	Prospective case–control	33 infants ≥ 36 weeks GA and 5 min Apgar scores ≤6 with severe asphyxia scores	33 infants ≥ 36 weeks GA and 5 min Apgar scores ≤6 with moderate asphyxia scores	SCr > 1.5 mg/dl	AKI occurred in 61% of those with severe asphyxia and 0% in those with moderate asphyxia	
Agras et al. (2004)	Retrospective chart review	45 term and preterm infants. Excluded cardiac	none	SCr > 1.5 mg/dl	Incidence; term = 3.4%	
					Premature infants = 31%	
					Non-oliguric = 40%	
					Asphyxia = 40%	
					Mortality = 24.4%	
Aggarwal et al. (2005)	Prospective case–control	25 infants ≥ 34 weeks, 5 min Apgar scores ≤6. Exclude those on diuretics or nephrotoxins	25 matched (GA and birth weight) infants	SCr > 1.5 mg/dl	AKI incidence: cases 56%, controls 4%	Clinical markers of asphyxia were better predictors of adverse outcome than were renal function tests
						Apgar scores ≤ 6 pick up most cases of renal failure
Gupta et al. (2005)	Prospective case–control	70 infants with 5 min Apgar scores ≤6	28 healthy controls	SCr > 1.5 mg/dl	AKI incidence: cases = 47.1%, controls 0%	78% of AKI non-oliguric
					Hospital mortality: cases = 14.1% vs ?	
Mathur et al. (2006)	Prospective case–control	52 infants with sepsis	146 infants with sepsis	BUN > 20 mg/dl on two occasions	AKI incidence: cases = 52/200 (26%)	Those with AKI more likely to have shock, DIC, meningitis and prematurity
					Hospital mortality: AKI = 70.2% vs no AKI = 25%	
Chevalier et al. (1984)	Descriptive observational	16 infants referred to pediatric nephrology service	none	SCr > 1.5 mg/dl	Hospital mortality = 25%	
					Non-oliguric renal failure had better outcomes	
Norman and Asadi (1979)	Descriptive observational	72/314 NICU admissions	none	BUN > 20 mg/dl and or UOP < 1 ml/kg per hour	Incidence of AKI 23%	
					52/72 responded to fluids	
					20/72 had no response to fluids	
					Mortality: 9/20 (45%)	

Two recent studies have explored AKI in the general critically ill neonatal population and confirm high incidences of AKI and deaths in these infants. In a retrospective analysis, Agras et al. [5] found a 25% hospital mortality rate in neonates with AKI. Many (47%) of their patients had non-oliguric renal failure and premature infants constituted 31% of the cases. This retrospective study is limited, because many infants did not have their SCr measured during potential AKI. Mathur et al. [8] prospectively studied mostly term neonates with sepsis and found a 26% incidence rate of AKI. The mortality rate was significantly higher in those with AKI than in those with no AKI (70.2% vs 25%, *P* <0.001). Although this study gives insight into the incidence of AKI in neonates, it is limited by the authors’ definition of AKI [blood urea nitrogen (BUN) > 20 mg/dl] and their inability to control for gestational age, birth weight, co-morbidities and severity of illness. Because of limitations in study design, one must use caution in making solid inferences about the true incidence and outcomes after neonatal AKI based on these studies.

Large observational studies of adults have convincingly shown that AKI independently predicts hospital length of stay, short and long-term survival in adults with AKI, once co-variables and complications have been carefully adjusted [12, 14, 15, 29–33]. In a meta-analysis, Ricci et al. [34] systematically reviewed all published articles on RIFLE that adjusted for co-morbidities and severity of illness. Compared with patients that did not have AKI, those with risk, injury, and failure had an adjusted relative risk (RR) of 2.4, 4.15, and 6.37, respectively (*P* < 0.0001 for all) (Table 4). Recently Akcan-Arikan applied AKI classification to children who required blood pressure or ventilator support and found that 80% had developed AKI under this classification. Those with a maximum RIFLE score for R had an adjusted odds ratio (OR) for death of 2.9 (0.8–10.9); those with RIFLE of I or F had an adjusted OR of 3.0 (1.1 to 8.1) [17].

**Table 4:** The RIFLE criteria and mortality rates for adults with acute kidney injury (adopted with permission from [34]) (*95% CI* 95% confidence interval)

Compared AKI levels	No. of studies	Adjusted RR	95% CI	*P* (overall effect)
Risk vs non-AKI	13	2.4	1.94, 2.97	<0.00001
Injury vs non-AKI	13	4.15	3.14, 5.48	<0.0001
Failure vs non-AKI	13	6.37	5.14, 7.90	<0.0001

One of the most common sequelae of prematurity is the propensity to develop bronchopulmonary dysplasia (BPD), as it affects 10% and 40% of surviving very low birth weight infants and extremely low birth rate infants, respectively [35]. The pathophysiology of this chronic lung condition involves elevated levels of pro-inflammatory interleukins, tumor necrosis factor-α (tnf-α), leukotrienes, and increased permeability of pulmonary vasculature, which culminate in abnormal lung development and fibrosis. Not only does AKI cause pulmonary edema secondary to volume overload, but also evidence from animal models of ischemic, nephrotoxic and bilateral nephrectomy shows that AKI induces a pro-inflammatory process highlighted by increased levels of neutrophils, tnf- α, interleukins, free radicals, endothelial growth factors and granulocyte colony stimulating factor [36–38]. Clinically, it has been recognized that not only do ventilated critically ill adults with AKI have a dismal prognosis (80% mortality rate) but they also have an impaired ability to be weaned from mechanical ventilation [31]. To date, little is known about the lung–kidney interactions in premature infants or the role the kidney has in BPD.

A large prospective cohort study with accepted definitions is greatly needed to help us better understand the incidence and independent impact of AKI on asphyxiated infants, premature infants, infants undergoing cardiopulmonary bypass and the general critically ill newborn populations. The role that the kidney plays in acute and chronic pulmonary diseases in premature infants needs to be explored.

## Novel early biomarkers of acute kidney injury

Because the incidence of AKI continues to rise, while the outcomes remain poor, nephrologists and intensivists continue to devote numerous resources for the better improvement of outcomes. Recent advances in the field of early AKI biomarkers have provided great optimism. Novel urine and serum biomarkers may change our approach to this condition if they can indicate AKI hours after an insult, in comparison with the days it may take serum creatinine to rise substantially. As opposed to our current functional markers of AKI (GFR and urine output), these biomarkers promise to signal injury early in the disease process, and, hopefully, they will allow us to intervene in the disease process at the onset of acute kidney ‘injury’ as opposed to attempting to fix acute kidney ‘failure’ [39, 40].

**Fig. 3:**
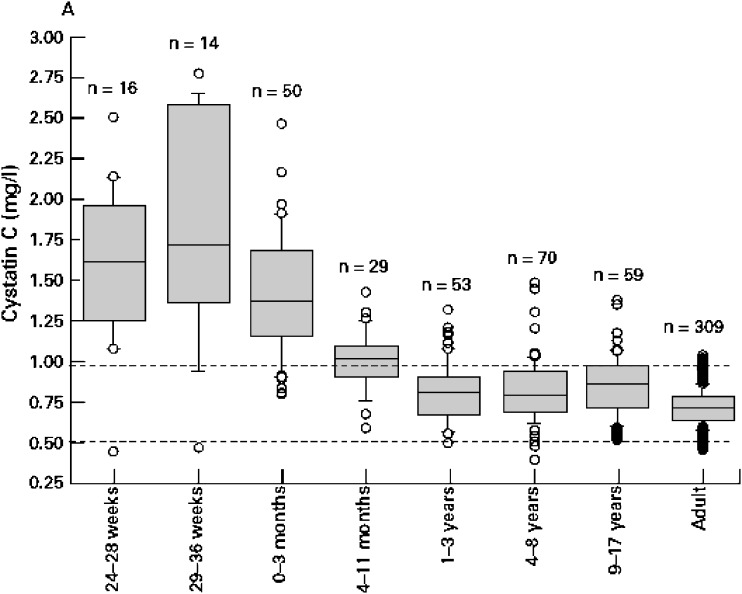
Box plot distribution showing cystatin C across age groups. The categories 24–28 weeks and 29–36 weeks refer to the gestational age of preterm babies. Preterm babies were 1 day old when the samples were drawn (reproduced with permission [45])

Biomarkers are currently being explored to differentiate between different causes of established AKI, to detect AKI early, and to prognosticate outcomes. Currently, the most promising early non-invasive biomarkers of AKI are serum and urinary neutrophil gelatinase-associated lipocalin (NGAL) [41], urinary interleukin-18 (IL-18) [42], kidney injury molecule-1 (KIM-1) [43, 44], and serum cystatin C [40]. Normal values in children are available [45] (Fig. 3). Many others continue to be explored, but they are beyond the scope of this review (see Coca et al. [46] and Nguyen and Devarajan [47] for reviews).

**Fig. 4:**
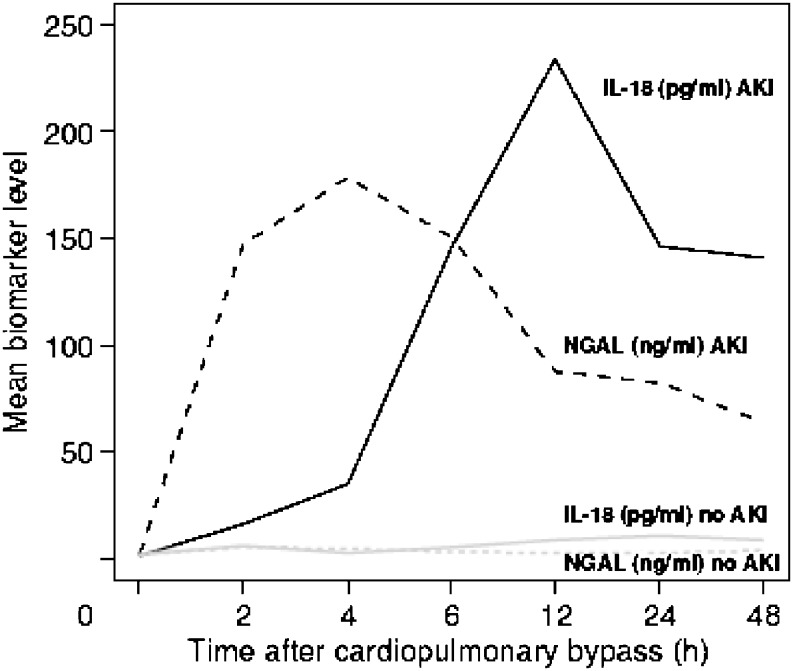
Pattern of urinary IL-18 and NGAL levels after cardiopulmonary bypass. AKI (defined as a > 50% increase in serum creatinine) developed after 48–72 h in the AKI patients (reproduced with permission from [49])

*NGAL* is one of the most strikingly up-regulated genes and over-expressed proteins in the kidney after ischemia [41]. Serum and urinary levels are elevated in human models of AKI, including neonates undergoing cardiopulmonary bypass surgery [48] (Fig. 4) and in a heterogeneous critically ill pediatric population [50, 51].

Lavery et al. recently examined 20 premature infants (divided into four birth weight categories with five subjects each) and looked at baseline urinary NGAL. They found that levels inversely correlated with both birth weight and gestational age. The wide baseline ranges narrowed over the course of 14 days [52], likely due to ongoing renal development. This finding is likely to be present for other biomarkers; thus, one of the challenges in our finding neonatal AKI biomarkers will be to account for the changes in renal developmental that will be present.

IL-18 urine levels are elevated in adults with ischemic AKI compared with other causes of renal injury, including urinary tract infection, nephrotic syndrome and chronic renal failure [49]. IL-18 levels are also elevated in neonates with AKI [42] following cardiopulmonary bypass (Fig. 4). In pediatric critically ill populations, IL-18 levels were significantly elevated early in those without sepsis and AKI, but not in those with sepsis [51, 53]. This highlights our need to test these biomarkers in different populations under different clinical scenarios.

NGAL, IL-18, KIM-1 cystatin C and many other biomarkers have been developed and are undergoing intensive testing in pediatric and adult critically ill populations. These non-invasive biomarkers of AKI need to be tested in the subsets of the critically ill neonatal population. In premature infants, baseline biomarkers may be changing during extra-uterine renal development, which will create additional challenges in the discovery of biomarkers of AKI in this population. Use of multiple markers together may better define the cause of AKI and provide information as to the time of injury. Improving our ability to diagnose AKI early in the disease process is an essential first step prior to implementing preventive and therapeutic interventions to improve outcomes.

## Neonates with acute kidney injury are at risk of developing chronic kidney disease

Premature infants have impaired glomerulogenesis which is more pronounced in those with acute kidney injury.

Using computer assisted morphometry, Rodriguez et al. measured radial glomerular counts (RGCs) in autopsied kidneys of premature infants with short vs long survival. They obtained the RGCs by counting the layers of glomeruli, following a straight line beginning in the deepest zone of the cortex and progressing systematically to the renal capsule, in well-oriented areas. They found that premature infants who had died early during their hospitalization had lower RGCs than term infants had. Premature infants who survived to the 40-week gestational (term) age had RGCs similar to those of premature infants with shorter survival, thus suggesting that the extra-uterine environment does not allow for proper neo-glomerulogenesis. In addition, infants with AKI had lower RGCs than did similar infants without AKI, suggesting that injury during this critical time can have substantial implications for total nephron counts [9]. Recently, Iacobelli et al. showed that, at a 6-year follow-up, premature infants with neonatal hypotension were at higher risk of microalbuminuria [54]. The authors were not able to correlate microalbuminuria to AKI, due to their study design.

### Low nephron mass leads to chronic kidney disease

The filtration rate of single nephrons and the number of nephrons present determine total GFR. When the number of nephrons is diminished, single-nephron GFR increases as the kidney works to compensate. This compensatory hypertrophy causes the glomeruli to function under increased intracapillary hydraulic pressure, which, over time, causes damage to the capillary walls. This abnormal process leads to progressive glomerulosclerosis, proteinuria, hypertension and chronic kidney disease [55]. The hyperfiltration hypothesis has been applied to, and confirmed in, autopsy data from hypertensive patients [56, 57] and in infants with intrauterine growth retardation (IUGR) [58–62].

### Acute kidney injury predicts/results in chronic kidney disease injury

Although most survivors of AKI have improved glomerular and tubular function prior to hospital discharge, long-term renal injury has been documented in children with congenital heart diseases [63], Henoch–Schönlein purpura [64], very low birth weight neonates [10] and hemolytic uremic syndrome [65]. The exact incidence of CKD after AKI is unknown, although growing data suggest that the incidence of CKD and mortality rates are high after AKI in adults [15, 66, 67]. We recently showed that, after pediatric AKI, the mortality rate is high in the years after hospital discharge. In addition, over 50% of children have at least one sign of CKD 3–5 years after the initial event [68].

Animal models have shown a clear relationship between AKI and long-term CKD caused by damage to endothelial vascular cells progressing to fibrosis and loss of function [69]. What is not known from these studies is the extent to which AKI leads to CKD in human populations, as it is unclear how often co-morbid conditions and pre-existing CKD are present before the hospitalization. Studying the long-term effects of AKI on CKD in neonates is ideal, as the causes of AKI and co-morbidities are more easily deciphered than in adults, where confounding diseases such as atherosclerosis, diabetes and smoking can all contribute to the events causing both AKI and CKD. Causal insights and strategies to prevent progression of CKD after AKI could have lasting effects on children and adults after AKI.

## Future directions

Numerous resources have been expended to improve our understanding of the pathophysiology, to test definitions and classification systems, and to develop non-invasive urinary biomarkers of AKI. Neonates have unique characteristics that challenge our ability to understand AKI in these populations and yet they are, in many ways, an ideal population to study because they lack the co-morbid and pre-existing problems that confound our understanding of how AKI affects outcomes. Because of the many shortfalls of using changes in SCr to diagnose AKI, the pediatric nephrology community needs to conduct studies to find better methods to diagnose neonatal AKI. Clear definitions which can predict hard endpoints need to be constructed. Epidemiologic studies to better understand the exact incidence and short- and long-term outcomes in this population are greatly needed. The kidneys’ role in systemic end-organ disease needs to be explored. Once these research questions have been answered, multi-center intervention studies to improve the short- and long-term outcomes in this vulnerable population can be performed.

## Self assessment questions: (Answers appear following the reference list)

1. Oliguria in term asphyxiated infants is a sensitive sign of AKI.
  - True
  - False
2. Asphyxiated infants with Apgar scores >7 are unlikely to have AKI.
  - True
  - False
3. Serum creatinine is not an ideal marker of AKI in neonates for the following reasons:
  - It reflects maternal creatinine in the first few days of life
  - There is a wide distribution, which varies by age and level of maturity
  - If the Jaffe method is used to measure creatinine, changing levels of bilirubin can alter reported levels
  - All of the above
4. Early biomarkers of AKI in neonates
  - Are unlikely to have any role in the diagnosis of neonatal AKI
  - Discovery will have additional challenges including differences in baseline levels of markers dependent on age and gestational age
  - Are currently being used to diagnose neonatal AKI early in the disease process
  - Cannot be used in urine due to the massive quantities needed for analysis
5. Premature infants with AKI should be followed for long-term consequences of CKD.
  - True
  - False
